# Intestinal microbiota, probiotics and mental health: from Metchnikoff to modern advances: Part II – contemporary contextual research

**DOI:** 10.1186/1757-4749-5-3

**Published:** 2013-03-14

**Authors:** Alison C Bested, Alan C Logan, Eva M Selhub

**Affiliations:** 1 Complex Chronic Diseases Program, BC Women's Hospital and Health Centre, B223A-4500 Oak Street, Vancouver, BC V6H 3N1, Canada; 2 CAMNR, 775 Blithedale Avenue, Suite 364, Mill Valley, CA 94941, USA; 3 Harvard Medical School and Massachusetts General Hospital, 40 Crescent St., Suite 201, Waltham, MA 02453, USA

**Keywords:** Intestinal microbiota, Autointoxication, Depression, Anxiety, Probiotics, Microbial ecology, Lipopolysaccharide endotoxin, Diet, Intestinal permeability, Microbial ecosystems

## Abstract

In recent years there has been a renewed interest concerning the ways in which the gastrointestinal tract – its functional integrity and microbial residents – might influence human mood (e.g. depression) and behavioral disorders. Once a hotbed of scientific interest in the early 20th century, this area lay dormant for decades, in part due to its association with the controversial term ‘autointoxication’. Here we review contemporary findings related to intestinal permeability, small intestinal bacterial overgrowth, lipopolysaccharide endotoxin (LPS) exposure, D-lactic acid, propionic acid, and discuss their relevance to microbiota and mental health. In addition, we include the context of modern dietary habits as they relate to depression, anxiety and their potential interaction with intestinal microbiota.

## Introduction

As outlined in Part I of this review, early 20th century scientists and clinicians placed emphasis on the importance of the gastrointestinal tract – its functional integrity and its microbes – as an influencing factor in depression and other mental health disorders. The interest in this topic, housed under the controversial term of autointoxication, disappeared rapidly by 1930. The reverse-order, top-down, focus would subsequently dominate research for decades; i.e. depression and anxiety as an influencing factor in the gastrointestinal disorders. We introduce Part II by reflecting upon one of the last articles of the autointoxication genera (1933), wherein neuropathologist Armando Ferraro and clinical psychiatrist Joseph E. Kilman of the New York Psychiatric Institute wrote the following in *Psychiatric Quarterly* journal [1]:

‘*It is far from our mind to conceive that all mental conditions have the same etiological factor, but we feel justified in recognizing the existence of cases of mental disorders which have as a basic etiological factor a toxic condition arising in the gastrointestinal tract*’

In 1933, with the understanding that ‘*we must not forget here the possibility that in the future more appropriate and more delicate biochemical methods may allow us to detect* [circulating gut-derived toxins] *in an easier and more accurate way than we are now able to do*’, Ferraro and Kilman proposed a multi-discipline collaborate effort. They wanted to establish a framework and begin with careful experimental lines of investigation. Their recommendation was to bring together experts to examine the following variables, while at the same time taking into consideration observable changes in animal behavior.

1. Alterations in intestinal permeability to toxins

2. The effect of synergy among potentially toxic chemicals arising from the gastrointestinal tract (e.g. others had only examined indole and p-cresol individually; in preliminary experiments, Ferraro and Kilman reported that gut-derived toxins produced CNS toxicity at much lower doses vs. solo administration).

3. Alterations to the intestinal microflora and factors of bacteriology

4. Alterations in liver and/or kidney function

5. Alterations to dietary patterns

Knowing that each of these variables could interact with one another, Ferraro and Kilman concluded ‘*All these problems will require time and we hope to be in a position to report on them some time in the future*’. Their paper and plans, however, lay dormant and virtually unreferenced. History would show that their research ‘wish-list’ would remain unfulfilled for 70 years. Here, in Part II, we will examine many lines of contemporary research and integrate what is now known concerning portions of Ferraro and Kilman’s framework. Threads from various branches of medicine, with the aid of advanced scientific technique, provide a mosaic of context that cannot be overlooked in future gut-brain-microbiota research. As we will discuss, the conundrum of interaction between the above variables continues to hamper the clinical relevance of experimental probiotic science in the brain and behavioral context.

### Part II – Modern studies of relevance to Gut-brain-microbiota connection

Within our original hypotheses on the potential value of probiotics for states of fatigue and depression [2,3], we sewed together separate lines of emerging research from multiple branches of medicine. The potential mechanistic pathways we proposed are highlighted in Table 1. Most noteworthy, perhaps, was the increasing recognition that mental health disorders are associated with low-grade inflammation (e.g. C-reactive protein), oxidative stress, and the elevation of inflammatory cytokines (e.g. TNF-α, IL-1, IL-6). Indeed, mood disturbances and fatigue could be induced by the systemic administration of lipopolysaccharide endotoxin (LPS) [4]. At the same time, with the emergence of publications indicating that probiotics could influence systemic cytokines, oxidative stress, and inflammatory markers upon oral consumption [5], a mood-regulating effect seemed scientifically plausible. There was also research around that time indicating that otherwise non-desirable gut microbes (e.g. *Campylobacter jejuni*), at quantities too low to produce a detectable immune response, could influence animal behavior indicative of human anxiety [6]. This suggested to us that probiotics, either directly, or through their influence on other gut microbes, may also play a role in precisely the opposite direction – benefits to cognition and behavior via gut-to-brain communication.

**Table 1:** **Probiotics for fatigue and depression - proposed pathways - adapted from *Medical Hypotheses*** - **Logan, et al.** [[2]] **and Logan, et al.** [[3]]

• Direct protection of the intestinal barrier	
• Influence on local and systemic antioxidant status, reduction in lipid peroxidation	
• Direct, microbial-produced neurochemical production – e.g. GABA	
• Indirect influence on neurotransmitter/neuropeptide production	
• Prevention of stress-induced alterations to overall intestinal microbiota	
• Direct activation of neural pathways between gut and brain	
• Limitation of inflammatory cytokine production	
• Modulation of neurotrophic chemicals including brain-derived neurotrophic factor	
• Limitation of carbohydrate malabsorption	
• Improvement of nutritional status – e.g. omega-3 fatty acids, minerals, phytochemicals	
• Limitation of small intestinal bacterial overgrowth	
• Reduction of amine/uremic toxin burden	
• Limitation of gastric/intestinal pathogens (e.g. *Helicobacter pylori*)	
• Analgesic properties	

In the decade subsequent to the hypotheses, the expansion of direct and indirect probiotic-brain research has been immense. A number of groups, including our own, have published preliminary human studies showing that orally consumed probiotic strains can indeed influence aspects of mental outlook and cognition [7-9]. The human studies have also been supported by a variety of elegant bench/rodent studies that have helped to shed light on the potential pathways whereby probiotic microorganisms can influence brain and behavior [10]. Below are updated discussions concerning these pathways and the critical links between the gastrointestinal tract, its microbial residents, and mental health. Although the gut-microbiota-brain connection may ultimately extend clinically to diverse conditions, from autism to schizophrenia [11,12], for the purposes of this article, the focus will largely remain on advances in depression and anxiety.

### Inflammation, oxidative stress and endotoxins

Within developed nations, up to one third of all visits to primary care clinicians involve patients with emotional disorders, most notably anxiety and/or depressive conditions [13]. As researchers explore the pathophysiology of mood and anxiety disorders, the links between elevations in markers of inflammation (e.g. cytokines, C-reactive protein) and emotional disorders have grown in strength [14]. Evidence also continues to indicate that the antioxidant defense system is overwhelmed in humans with major depressive disorder and that the burden of oxidative stress and inflammation becomes a viscous cycle [15]. Remarkably, even skin biopsy samples from depressed patients (vs. matched healthy controls) show higher levels of oxidative stress [16]. Moreover, researchers are beginning to understand how and why inflammation and oxidative stress can be both a cause and a consequence of depression [17]. Elevation in systemic inflammatory cytokines can signal the production of inflammatory cytokines within the central nervous system via microglia activation. Despite their critical role in neuronal repair, chronic activation of microglia can compromise neuronal functioning by setting in motion a cascade of inflammation and oxidative stress [18]. This, in turn, can compromise normal intra and extracellular neuronal communication.

Although a number of gut-derived bacterial products, Staphylococcal enterotoxin B as one example, can induce behavioral changes indicative of anxiety when administered in the periphery, a key component of the gut microbiota to brain connection are the increasingly robust studies involving the systemic administration of LPS endotoxins. LPS administration at low levels (e.g. 0.4ng/kg) has been shown to cause acute anxiety, depressive symptoms, cognitive deficits and increased visceral pain sensitivity [19-23]. The LPS is a structural portion of the external membrane of gram-negative bacteria, and it is estimated that humans harbor at least a gram of LPS in the intestinal lumen. In healthy adults there may be miniscule amounts of LPS found in blood plasma (1-200pg/ml), indicating that the intestinal barrier performs its exclusionary role with good efficiency [24]. However, higher circulating LPS in the healthy general population and patient groups is positively associated with abdominal obesity, higher insulin, triglycerides, total cholesterol and other markers of cardiovascular and diabetes disease risk [25-27]. LPS is cleared from circulation by the liver and its enzyme alkaline phosphatase (AP) in particular [28]. LPS up-regulates AP activity and induces oxidative stress in the liver, therefore the ways in which antidepressant agents influence alkaline phosphatase and liver detoxification is under increased scrutiny [29].

Experimental investigations have made it clear that LPS-induced peripheral cytokine elevations can alter neuronal activity in the limbic system (e.g. increased amygdala activity) [30,31]. As well as causing excitotoxic neuronal overstimulation, the LPS-induced inflammation can increase the activity of indoleamine-2,3-dioxyegenase (IDO), an enzyme that breaks down tryptophan in the kynurenine pathway. IDO activity has been positively correlated with depressive symptoms, and kynurenine itself can increase anxiety when administered in the periphery. The end result of endotoxemia may be decreased tryptophan and enhanced kynurenine availability, along with compromised serotonergic functioning [32-34]. On the other hand, kynurenic acid, produced by commensal intestinal microbiota and found in traditional foods (honey, green vegetables, tubers), is readily absorbed from the GI tract [35,36], and has been reported to have anxiolytic activity when administered in the periphery [37]. Systemic LPS can also induce brain or CNS cytokine production and alter behavior without the necessity of systemic cytokine involvement, with some research indicating a direct LPS to CNS communication via brain endothelial cells [38]. Indeed, systemic LPS can compromise the integrity of the normal blood–brain barrier (BBB) and facilitate the passage of a host of potential agents (environmental toxins, pathogens etc.) as well as LPS itself [39]. To make matters worse, the ability to remove potentially harmful neurotoxins (e.g. amyloid beta-derived neurotoxins) from the brain through normally functioning efflux mechanisms at the BBB is also compromised by LPS [40]. Among those with depression, the subsequent risk of dementia or mild cognitive impairment is up to 2-fold higher, and researchers continue to evaluate low-grade inflammation as a primary driver of cognitive decline [41]. Permeability of the blood–brain barrier and the intestinal barrier is, at least in part, controlled by endocannabinoid modulation. Endogenous cannabinoids are increased during inflammation (as are their receptors) in what appears to be an adaptive process - cannabinoid binding to receptors can attenuate LPS-induced blood–brain barrier permeability and intestinal inflammatory effects of LPS [42,43]. It is also worth noting that in animal studies, previous experience with physical and/or psychological stress (e.g. social defeat, tail shock) leads to an even more pronounced inflammatory cytokine release subsequent to LPS administration [44]. This suggests that in clinical populations certain individuals may be primed to experience a greater burden of inflammation and oxidative stress when specific agents (environmental toxins, bacterial products, etc.) find their way into systemic circulation.

### Intestinal permeability, bacterial overgrowth

Of course, an obvious pathway to increased LPS exposure in the periphery would be a compromised intestinal barrier, and recent investigations have indeed shown signs of increased intestinal permeability in depression. In findings reminiscent of Johnson and Goodall’s 1904 report on serum *E. coli* agglutination in depression [45], new studies report that patients with major depression (vs. healthy controls) have higher serum IgM and IgA against a variety of commensal gut bacteria [46-48]. Moreover, in conditions where depressive symptoms are often part of the picture – irritable bowel syndrome (IBS), myalgic encephalomyelitis (ME, aka chronic fatigue syndrome), fibromyalgia (FM), complex regional pain syndrome, alcohol dependency, insulin resistance, and obesity – studies involving human subjects have shown high rates of intestinal permeability [49-53]. Ferraro and Kilman were most intrigued why, in their experiments, certain animals of a given species were more resilient to the systemic consequences of intestinally-derived toxins than same-species counterparts [1]. Controlling for body weight, why didn’t all animals react in similar fashion to equivalent systemic doses of gut-derived toxins? Seventy-seven years later, Italian researchers would show that abnormal intestinal permeability (IP) is a common finding in first-degree relatives of those with autism (36.7% abnormal IP in those with autism, 21.2% in first-degree relatives, 4.8% in healthy controls) [54]. Backing up the claims of Fenton Turck over a century ago [55], psychological stress and splanchnic hypoperfusion subsequent to exhaustive exercise have recently been shown to increase intestinal permeability in adults [56,57]. Obviously the finding of increased intestinal permeability in over-lapping medical conditions provides clinical meaning to the LPS discussions above. In addition to LPS, a more porous intestinal barrier allows systemic access to food antigens (e.g. gliadin) environmental toxins (e.g. polychlorinated biphenyls etc.), exposure to which is a further risk factor for depressive symptoms [58,59]. Polychlorinated biphenyls can further compromise the integrity of the intestinal lining and cause deficits to the normal BBB permeability [60,61]. Meanwhile, probiotics have been shown to influence removal of environmental toxins from the gastrointestinal tract [62].

The early reports of low gastric acid among patients with melancholia, discussed in Part I, have been confirmed by more modern investigations involving patients with major depression [63,64]. The consequences of low stomach acid production include small intestinal bacterial overgrowth (SIBO) and further damage to the intestinal barrier. Use of gastric acid-blocking medications can provoke SIBO [65]. Clinically, SIBO sits on a wide continuum from asymptomatic to a severe malabsorption syndrome. For many, there may be very mild gastrointestinal symptoms, including bloating, diarrhea, abdominal pain, and constipation [66]. Once again, much like intestinal permeability, SIBO has been found in the aforementioned conditions - IBS, ME, FM, insulin resistance, and obesity [67-70]; it is strongly associated with depression and anxiety [71]. Interestingly, SIBO is found in erosive esophagitis [72], yet in the prospective treatment of gastro-esophageal reflux disease, those with depression and/or anxiety are the least likely to respond to proton pump inhibitors [73]. Given that SIBO and intestinal permeability have been linked together, an over-lap among these medical conditions should not be surprising. Remarkably, eradication of SIBO with antimicrobials improves emotional symptoms and restores the normal intestinal barrier [74]. SIBO can compromise proper absorption of proteins, fats, carbohydrates, B vitamins, and other micronutrients due to bacterial interference. Excess bacteria can successfully compete for nutrients, produce toxic metabolites, and cause direct injury to enterocytes in the small intestine [75,76]. All of this could, of course, influence mood indirectly. On the other hand, experimental studies show that psychological stress stagnates normal small intestinal transit time, encourages overgrowth of bacteria, and compromises the intestinal barrier [77]. In animal research, diarrhea and intestinal permeability are also reported together in the context of SIBO and stress [78]. The oral administration of probiotics has been shown to be beneficial in the reduction of SIBO [79]. Experimentally, an omega-3 deficient diet increases SIBO, an interesting finding given the consistent relationship between inadequate omega-3 and depression [80].

### Intestinal microbiota, diet and modernization

With an appreciation of inflammation, oxidative stress, LPS, intestinal permeability and SIBO as background, we can step back and examine the relevance of modern dietary patterns in the context of gut to brain health as mediated by microbes. Regarding the broad environmental influences on mental health, it has been shown in preliminary research that richness in species biodiversity (e.g. vegetation and birds) within urban environments is positively associated with mental well-being [81,82]. The same likely holds true regarding microbial biodiversity within the gut. For example, a loss of bacterial biodiversity is associated with GI disorders, skin conditions and obesity [83]. Remarkably, a recent study showed that diminished environmental biodiversity (measured as richness of a wide variety of plant species) in the proximity of personal residence is associated with lower genetic biodiversity of a bacterial species (gammaproteobacteria) on skin samples of those with atopy. This bacterial species and its upstream genus *Acinetobacter* have been specifically linked to anti-inflammatory cytokine production [84]. It is becoming increasingly clear that indigenous or traditional dietary patterns are inclusive of many bacterium species that might be considered to have probiotic potential. Consider that an estimated 35% of all lactic acid bacteria isolated from raw fruits and vegetables can survive gastric conditions [85]. In the 1980s researchers made note of some significant differences in the fecal microflora of rural Japanese vs. Canadian urbanites. They found higher counts of bifidobacterium spp. and lactobacilli in the rural Japanese who maintained a traditional high-fiber diet rich in fermented foods, vegetables and fish. Using culture technique, Benno reported higher amounts of clostridia spp. in the Canadians, yet overall, there was greater biodiversity (more genera and species) in the rural-dwelling Japanese [86]. Following up, his team reported on the differences in fecal microflora among older adults residing in Tokyo vs. elderly rural Japanese maintaining a high-fiber traditional diet. Once again, there were higher numbers of bifidobacterium spp. among the rural dwellers and lower amounts of clostridium spp, particularly *C. perfringens*[87].

More recent investigations have also shown marked differences in the fecal microbiota of western European children vs. rural African children living in an environment resembling that of Neolithic subsistence farmers. Using DNA sequencing of samples, researchers reported less potentially pathogenic bacteria, yet a far greater degree of biodiversity and microbial richness in rural Africans living a traditional lifestyle and consuming traditional high fiber foods [88]. A separate group of researchers has also noted distinct differences in the bacterial groups and their functional genes (those involving metabolism of amino acids, etc.) in US adults living in metropolitan centers vs. villagers living in Africa and South America. Once again, the fecal microbiota of US metropolitan adults showed far less diversity vs. villagers in these distinct regions. Diet was reported to be the control switch for intestinal microbiota, trumping other influences such as hygiene [89]. Beyond the ‘hygeine hypothesis’ (lack of exposure to diverse microbes increases risk of allergies and exaggerated immune response), researchers are questioning the broad implications of loss of microbial diversity as a consequence of modernization. DNA sequencing of stool samples in combination with detailed dietary analysis have allowed researchers to determine that long-term dietary patterns largely determine the main phyla of the gut microbial profile [90]. However, even short-term dietary changes can induce species-level changes to the intestinal microbial residents, as can psychological stress [91]. Although the administration of beneficial microbes may not have a major impact on stable phyla, probiotic intervention studies (as discussed later) serve to remind scientists that species-level application of microbes cannot be overlooked as clinically relevant. A single strain of lactobacillus can improve overall microbial diversity [83], while the administration of a single bifidobacterium strains can increase the quantity of separate bifidobacterium species and overall lactobacilli within the gut [92,93].

To date, detailed analyses of the gut microbiota of patients with major depressive disorder have not been conducted by contemporary investigators. However, it is worth noting that alterations to ‘normal’ (i.e. modernized) human gut microbiota have been observed in the same group of aforementioned chronic conditions associated with a high degree of depressive symptoms – IBS, ME, obesity and type 2 diabetes [94-98]. A growing number of studies in both animals and humans are continuing to highlight the divergent effects of diet (fast-food style Western diet vs. traditional dietary patterns) on microbiota, LPS burden, and intestinal permeability [24]. Studies show that the high-energy (high-fat, low-fiber carbohydrates) diets typical of Western nations, can diminish levels of bifidobacteria, increase intestinal permeability and elevate blood LPS levels [99]. Human research shows that adherence to a typical Western-style diet (high fat and low-fiber carbohydrates) for one month can elevate plasma endotoxin activity by 71%. By contrast, a switch to a low-fat, low saturated fat, fiber-rich diet (so-called ‘prudent’ or more traditional diet) for one month can decrease baseline blood endotoxin activity by 38% in healthy adults [100]. A separate 24-week study, in adults with clinical and laboratory signs of metabolic syndrome, showed that a low fat and high carbohydrate diet increased fecal bifidobacterium and reduced fasting glucose compared to baseline [101]. Animal studies indicate that when fat intake is very high (72%) there is a 2.7-fold increase in circulating LPS, while diets with 40% fat elevate circulating LPS by 1.4-fold [102]. Recall that circulating LPS can interfere with BBB function, therefore it should not be surprising that high fat (40% fat, mostly saturated fat from lard) diet disturbed cognitive performance and increased BBB permeability. Compared to the control standard chow group, the researchers discovered that the high-fat diet allowed a systemically administered dye, one normally excluded from the brain, into the hippocampus [103]. Separate groups of researchers have reported similar findings of a compromised BBB when animals are placed on Western-style diets [104].

The implications of this research to neuropsychiatric disorders are immense. With this as background, and given the focus on depression in the current paper, it is noteworthy that multiple epidemiological studies show that adherence to healthy dietary patterns, particularly the Mediterranean diet (similar to the prudent diet), is associated with lowered risk of depression and anxiety [105-108]. The reduction in risk of depression is clinically meaningful; consider that in 5-year prospective research, the resiliency factor of adherence to a Mediterranean and traditional dietary patterns is between 25-30% [107,108]. However, patients with depression and fatigue are typically far removed from traditional, prudent diets. Indeed, stress, depressive symptoms and high levels of anxiety are characteristically associated with increased frequency of fast-food choices and over-consumption of high-energy, low nutrient density foods [109-111]. Among the many implications of this dietary pattern in mental health, its ability to establish an ‘inflammatory microbiome’ is now worthy of further investigation.

To further illustrate the relevance of dietary choices in the entire gut-brain-microbiome spectrum, consider that more detailed analysis reveals that not all types of fats and/or simple carbohydrates influence these end-points (microbiota, intestinal permeability, LPS) by equal measure – for example, omega-3 oils, vs. oils rich in saturated fats, may have a positive influence on the intestinal barrier and limit the detrimental effects of LPS [112-114]. Conversely, the administration of bifidobacteria is associated with higher omega-3 levels in brain cells [115]. In other words, to what extent are the reported mental health benefits of omega-3 fatty acids determined by resident intestinal microbiota? Among the simple sugars, fructose administration has been shown to increase circulating LPS by 40% (vs. controls, sucrose and glucose). In this dietary sugar research, the LPS was normalized after the administration of an antibiotic, indicating a direct microbial influence [116]. Given the massive rise in high-fructose corn syrup (now representing 42% of all caloric sweeteners) [117], surely we must question its role in epidemiological studies linking overall simple sugar consumption to depression [118,119]. LPS and the microbiota could certainly be connected to the fairly robust relationship between fructose intolerance and depression [120,121]. Lowered blood tryptophan levels have been observed in fructose intolerance [122]; however, the new findings on LPS are a clear indication for further research.

The intestinal microbiota may also be involved in the potential value of dietary antioxidants to brain health. Fiber-rich carbohydrates, including whole fruits and vegetables, are abundant in a variety of antioxidant phytochemicals, most notably polyphenols. Researchers are currently examining the complementary ways in which antioxidant-rich foods and beverages influence gut microbiota and, in turn, how select constituents of gut microbiota influence phytochemical absorption [123-127]. For example, major dietary antioxidant sources such as cocoa, coffee, green tea, blueberries, and curcumin have all been linked in epidemiological studies to lowered risk of depression and/or cognitive decline [128-132]. Meanwhile, these dietary agents have been shown in human and animal studies to have beneficial effects on gut microbiota, including promoting the growth of lactobacilli and bifidobacteria [133-137]. Curcumin was recently shown to prevent LPS-induced intestinal permeability in an experimental model, while green tea reduces LPS-induced sickness behavior and BBB permeability [138-140]. Consider also the previously mentioned research on high-fat/high-sugar (fructose) diets and elevated circulating LPS. In human research the consumption of orange juice along with a high-fat/high-carbohydrate (low fiber) meal prevented elevations in circulating endotoxin, elevations that were otherwise observed when the same meal is consumed with either pure water or glucose in water at the same concentration as found in orange juice [141]. Despite its fructose content, orange juice may negate the detrimental effects by virtue of its rich content of flavonoids.

Experimentally the links between these dietary influences on microbes (or microbes on the absorption of dietary components) and mood are evident; consider, for example, the observations that blueberry species, green tea, curcumin, pomegranate, and resveratrol polyphenolic extracts exert antidepressant activity and stress resiliency in animal models [142-146]. Research indicates that once absorbed, single phenolic structures can have subtle but meaningful effects on the breakdown of central neurotransmitters [146]. Emerging studies also allow for the suggestion that the behavioral benefits might be explained, at least in part, by their phytochemical influence on minimizing the effects of LPS and promoting lactobacilli and bifidobacteria growth at the expense of potentially pathogenic microbes. For example, long-term intake of honey, a feature of Paleolithic Age and traditional diets, has been shown in animal research to be associated with decreased anxiety and cognitive decline vs. sugar-free and sucrose controls [147]. It is worth noting that honey, in addition to its broad array of antioxidant phytochemicals, has been shown to negate the effects of LPS and increase the growth of lactobacilli and bifidobacterium [148,149].

Lactic acid bacterium-inclusive (non-dairy) fermented foods are an important part of traditional Asian dietary patterns, and some of the Lactobacillus strains within these foods beverages and herbs, most notably *L. plantarum*, have been shown to have significant antioxidant activity, prevent intestinal permeability, and lower the stress-induced LPS burden [150,151]. Fermented rice products have shown antidepressant and anti-fatigue effects vs. standard rice in experimental animal models [152]. In controlled human research, *L. plantarum* 299v has been shown to improve pain in adults with IBS, and the same strain can reduce markers of systemic oxidative stress by 37% and markers of inflammation by 42% in adult smokers [5,153]. In animal studies maternal *L. plantarum* administration during pregnancy (and to offspring in early life – i.e. 6 months) increased microbial diversity and had anti-obesity effects later on in the life of offspring [154]. *L. plantarum* 299v, *L. plantarum* DSM 15313 and other probiotics are currently being investigated for their ability to enhance the absorption of active polyphenolic chemicals that would be of relevance to brain health [155,156]. It is not our desire to belabor the point concerning the interaction between food and microbes; however, there are countless other components of traditional diets that could be singled out for relevancy. The seeds of Nigella sativa (black cumin), a component of traditional dietaries in parts of Africa and South Asia, provide a clear example. Nigella sativa has been shown to protect the intestinal mucosa, suppress the growth of potentially harmful gut microbiota, and prevent LPS-induced depression-like behavior in animals [157-159]. Consider also that naturally fermented goat milk in traditional diets is higher in the protein lactoferrin [160], yet another agent that promotes the growth of bifidobacterium at the expense of potentially pathogenic microbes and one that can also minimize the detrimental effects of LPS [161-163]. Oral lactoferrin reduces systemic oxidative stress, and two week consumption of lactoferrin-rich colostrum has been shown to attenuate the intestinal permeability induced by exhaustive exercise in healthy athletes [164].

Even individual nutrients such as magnesium, a mineral found richly in green plant foods and the intake of which has been reported to be low in depression [165], may be a factor. In experimental studies a magnesium-deficient diet is associated with intestinal permeability and quantitative changes to cecal bifidobacteria and lactobacilli [166]. Zinc is yet another nutrient consistently linked to depression when long-term intake is sub-optimal [167], and once again it is a nutrient that can influence the diversity of the intestinal microbiota and intestinal permeability [168]. Magnesium and zinc are necessary for the activity of intestinal alkaline phosphatase (IAP), a critical enzyme involved in preserving the diversity of gut microbiota and decreasing the systemic LPS burden. IAP production itself is kept in balance by the diversity of intestinal microbiota [169,170]. The emerging research might therefore suggest that at least one bridge between healthy traditional diets and mental health includes resident microbiota. The research discussed below in the following sections of our series should only serve to strengthen this view.

### D-lactic and propionic acid – a cautionary tale

Beyond the general findings of lower bifidobacteria and lactobacilli, a study involving ME (chronic fatigue syndrome) patients provides one of the most interesting and unexplored links between gut dysbiosis and mental health. Specifically, these researchers found significantly higher levels of enteroccocus and streptococcus spp. in the fecal samples of ME patients vs. controls. In particular, *E. faecalis* and *S. sanguinis* were found in higher numbers, and upon closer examination it was found that these two species manufacture significantly more D-lactic acid upon glucose exposure [98]. Although a variety of microbes in the gut, including probiotics, are also known to produce lactic acid (L- and D- isomers), it is the elevated production of D-lactic acid that becomes a concern. D-lactic acid is broken down much more slowly than L-lactic acid, and when it begins to accumulate in the intestine it can push absorption into the blood. Other factors can enhance D-lactic acid absorption into the blood, most notably, LPS administration, intestinal permeability, psychological stress, and osmotic pressure [171,172].

Clinically, significant D-lactic acid accumulation (i.e. that leading to metabolic acidosis) is largely viewed as a rare concern, one primarily associated only with short bowel syndrome. However, with the new findings reported in ME, and separate animal studies on subtle effects of gut-derived D-lactic acid, researchers are reframing the relevance of D-lactic acid producing gut microbes and carbohydrate fermentation. Indeed, animals with excessive carbohydrate fermentation and D-lactic acid production in the cecum exhibit anxiety, aggression and impaired memory [173-175]. The latter study also investigated plasma D-lactic acid and associated the cognitive impairment with increasing blood levels of D-lactic acid. It is noteworthy that D-lactic acid can move through the BBB and appears to interfere with neuronal energy supply via limitation of pyruvate availability. These novel studies involve low-grade D-lactic acid levels in the periphery, well below that observed in overt lactic acidosis, and the findings could be the link between contemporary SIBO, LPS, intestinal permeability and the historical ‘enterosthenia’. It is entirely possible that SIBO and carbohydrate intolerance (excess undigested carbohydrate arriving at the colon) can drive D-lactic acid production, absorption, and subsequent alterations in perception of fatigue, aspects of cognition and behavior. At least one experimental study has shown that bifidobacterial surface proteins can prevent intestinal permeability and elevations in blood D-lactic acid levels subsequent to ischemia [176].

This emerging research would also suggest that selection criteria of probiotics for use in mental health clinical trials should include strains with higher L- vs. D-lactic acid production (e.g. strains of *L. delbrueckii* are notorious producers of D-lactic acid) [177,178]. Moreover, the emerging research on D-lactic acid, as induced by excessive fermentation, might also serve to warn investigators about the downsides of too much of a good thing – i.e. too much prebiotic fiber could encourage the growth of high D-lactic acid-producing species. Experimental and human research shows that excess prebiotic fiber can be an irritant to the gut barrier, increasing intestinal permeability [179]. Recently beta-glucans, often touted for prebiotic activity, were shown to increase intestinal permeability in an ex vivo animal study [180]. These findings should be taken into consideration in clinical trials regarding patient selection, and in particular those who might report carbohydrate intolerance or worsening of GI symptoms upon bran or other types of fiber consumption should be differentiated as part of trial designs. Although it is often assumed that the elevation of intestinal propionate production via prebiotics is a beneficial to the host, it is worth pointing out that systemic propionic acid reduces locomotor activity, and when it enters the brain it can induce a neuroinflammatory response and impair social behavior in animals [181]. In short, the well-meaning lay advice to supplement with prebiotics, or the wrong types of probiotics, may have unintended consequences to sub-groups of patients within the modern day ‘entersothenia’ spectrum. It is entirely possible that large doses of prebiotics or strains of *L. delbrueckii* may *increase* anxiety in those with, for example, co-morbid IBS and anxiety.

### Lessons from germ-free animals

The original work of Cohendy in 1912, wherein he demonstrated the resiliency of chickens reared in a germ-free environment [182], takes on new meaning with emerging studies involving germ-free mice. Studies have recently shown that mice reared in germ-free environments (consuming only autoclaved food) actually displayed decreased anxiety vs. animals raised with an intact intestinal microbiota (one without specific pathogens). The behavioral changes were linked to some distinct differences in neurotransmitter turnover, as well as genetic and protein expression for receptors and neuronal plasticity [183]. Moreover, the transfer of conventional microbiota to germ-free animals early in life seemed to negate the behavioral/neuronal differences, while transfer in adulthood did not reverse the behavioral signs of less anxiety in the originally germ-free animals. Other studies have reported similar findings [184-186]. One interpretation of the research is that throughout evolution, colonization with gut microbiota in early life became a critical mediator of some aspects of brain development; i.e. communication from gut-to-brain via microbes helped facilitate a higher level of subsequent vigilance and threat-based surveillance, particularly in our ability to read commensal shifts and/or the presence of potentially pathogenic microbes. Experimentally, germ-free animals have a compromised ability to form memories [187], and this too may reflect lower baseline vigilance. Recall the earlier discussion regarding low levels of circulating LPS among healthy individuals, therefore it is plausible that even very low levels of LPS (sampled via “normal” microbiota) may act as a kindling to increased vigilance in the conventional animals. Indeed, gut commensal bacteria can efficiently detect the presence of a parasite (*Toxoplasma gondii*) in the intestine, and in turn, initiate signals for a defensive immune response – all this without the parasite actually activating specific receptors otherwise responsible for the detection of infectious pathogens [188].

The behavioral signs of decreased anxiety in germ-free animals are not necessarily a good thing, at least not in clinical terms. The experimental studies to date have mostly utilized the elevated plus maze (EPM) as the primary tool in objective behavioral assessment. Briefly, the EPM is a plus-sign-shaped apparatus that is typically set up around 2 feet off the laboratory floor; it includes two open arms (extending planks) without walls and two closed arms with walls and an open top. Exploration out from the closed areas to open arms is taken as a behavioral sign of decreased anxiety in an animal. In controlled studies the EPM is suitable in efforts to determine if an intervention (e.g. medication) can influence approach/avoidance behavior in animals, although even here its universal acceptance as a means to specifically and objectively measure anxiety has recently been called into question [189]. In the germ-free rodent studies, assuming that the mice with the healthy gut microbiota are the “otherwise well-adjusted” animals exhibiting normal baseline behavior, exploration in the open arms by the germ-free mice can also be taken as a sign of increased risk-taking; it is entirely possible that a microbe-free gut encourages a higher level of risky behavior, one that might put the animal in harm’s way of a predator. In one of the studies [183] the germ-free mice explored to the extreme ends of the open arms almost times more often than did the conventional mice, certainly suggesting a higher degree of risky behavior.

Therefore, the question remains, to what extent is the commensal bacteria protecting us? To what extent does it provide support in the development of an appropriate level of vigilance? Recall the importance of normal, healthy intestinal microbiota in launching a defense against *T. gondii*[188]. Potential relationships between *T. gondii*, alterations to normal intestinal microbiota, intestinal permeability, behavioral risk-taking in animals, human schizophrenia, suicide risk, and depression [11,190,191] is an area worthy of investigation. How would germ-free animals react to predator odor? The amygdala is a critical structure for legitimate threat detection and vigilance, and lesions of the amygdala in primates and humans extinguish the aversion to monetary loss [192]. Germ-free mice were noted to have decreased N-methyl-D-aspartate (NMDA) receptor mRNA expression in the central amygdala, leading to speculation of decreased amygdala activity in concert with decreased anxiety [183]. It is noteworthy that activity in the central amygdala is involved in processing the reaction to predator odor [193]. Moreover, in one study involving germ-free mice, confinement stress produced higher levels of plasma corticosterone in the germ-free animals vs. those with pathogen-free microflora and in those where the gut was inhabited by *bifidobacterium infantis*[184]. In humans, a large cortisol response under stress is associated with decision making that is in line with immediate reward and high risk of punishment. In rodents glucocorticoid elevation predicts self-administration of psychostimulant drugs and evidence suggests that cortisol itself may be a driver of highly rewarding behavior at the expense of contemplation of risk [194].

Recently investigators have examined the effects of a Western diet (higher dietary fat, cholesterol, sugar) consumed by mice during pregnancy and determined that changes to milk quality induces systemic inflammation in the offspring. The inflammation and toxicity among pups nursed by Western diet-consuming mothers was even more pronounced when the parent was also raised as germ-free [195]. This highlights that certain commensal bacteria may afford a layer of protection against the effects of a Western-diet during a delicate developmental stage. The research also highlights that the relationships between diet, microbiota, metabolism, inflammation and behavior are intertwined. Reductionist approaches have certainly uncovered that microbiota alone can act as a CNS-influencing entity; however, there are a host of drawbacks in using germ-free mice, or rodents in general, to speculate on complex human gut-brain communications. Consider that shortly after germ-free mice are colonized with just a single species of commensal bacterium, researchers note marked changes to the expression of genes that govern intestinal permeability, nutrient absorption, blood vessel growth, and the metabolism of environmental toxins [196] – all of which could influence behavior via LPS and other pathways. A single strain of bifidobacterium, for example, can elevate blood tryptophan levels in animals, meanwhile tryptophan depletion during lactation can interact with stress hormones to increase anxiety later in life [197].

Interesting studies with germ-free animals have little to no clinical frame of reference in mental health - i.e. humans do not live with a sterile GI tract while consuming exclusively autoclaved food; however, they do indicate that developmentally our resident microbiota, or the absence thereof, can have a long-standing influence on brain functioning and behavior. Researchers might ponder if long-term mental health consequences could be associated with the routine prophylactic use of broad-spectrum antimicrobials among pre-term infants. This practice, one that has been questioned recently [198], sets up a pseudo-germ-free situation in premature infants, and it can now be yet one more factor to consider in the link between pre-term birth and mental illness. Recently, a large epidemiological study using the Swedish Medical Birth Register – 1.3 million people - has found that premature birth is associated with a 30% higher risk of depression and a 270% increase in bipolar depression later in life [199]. At some point in the future, with more detailed investigations, the germ-free studies may be of relevance in bridging the research on the immune benefits of prenatal exposure to probiotics, and on the other side, the increased risk of serious mental illness (in adult offspring) subsequent to infection during pregnancy [200]. Of course, there is also the issue of intestinal microbiota and their effects on the elimination of environmental toxins. When animals are exposed to low doses of bisphenol A (BPA) during gestation, lactation and nursing, there are subsequently marked increases in later-life anxiety accompanied by alterations in gene expression within the amygdala [201]. With the recent discovery that oral probiotics co-administered with BPA can dramatically reduce the systemic burden of this and other environmental toxins (increasing elimination via fecal route), it becomes difficult to view the ‘real world’ gut-brain connection in germ-free isolation. The influence of probiotic administration on both the developing immune system and metabolism during the introduction of foods (weaning diet) is an exciting area of research, with preliminary results indicating long-term systemic effects [202].

### The stage is set

The research discussed here in Part II can be considered contextual; before examining more clinically relevant investigations on behavior and microbiota, it seems vital to underscore the fact that any discussion of microbiota and mental health, or probiotics as an intervention in behavioral medicine, is not akin to discussions of alprazolam, fluoxetine, etc. There are a myriad of ways in which the microbes of the intestinal tract may interact with environmental factors, most notably those of the dietary. As described here in Part II, some of Ferraro and Kilman’s 1933 ‘wish list’ of research has been initiated, including the effects of intestinal permeability and dietary alterations in relation to microbes and mental health. In Part III we will turn our attention to the other aspects, including purposeful alterations of the intestinal microbiota (dysbiosis), the unresolved effects of intestinal-derived toxins, and the experimental and clinical application of factors of bacteriology (probiotics).

## Competing interests

ACB and EMS have no competing interests. ACL has received consulting fees from Genuine Health, Toronto, Canada.

## Authors’ contributions

ACB, ACL and EMS contributed equal time and effort in the investigation, research and drafting of this manuscript. All authors read and approved the final manuscript.
